# Design, Synthesis, and Investigation of Novel Nitric Oxide (NO)-Releasing Aromatic Aldehydes as Drug Candidates for the Treatment of Sickle Cell Disease

**DOI:** 10.3390/molecules27206835

**Published:** 2022-10-12

**Authors:** Boshi Huang, Mohini S. Ghatge, Akua K. Donkor, Faik N. Musayev, Tanvi M. Deshpande, Mohammed Al-Awadh, Rana T. Alhashimi, Hongmei Zhu, Abdelsattar M. Omar, Marilyn J. Telen, Yan Zhang, Tim J. McMahon, Osheiza Abdulmalik, Martin K. Safo

**Affiliations:** 1 Department of Medicinal Chemistry, The Institute for Structural Biology, Drug Discovery and Development, School of Pharmacy, Virginia Commonwealth University, Richmond, VA 23298, USA; 2 Vertex Pharmaceutical Incorporated, Boston, MA 02210, USA; 3 Department of Medicine, Duke University Health System, Durham NC 27710, USA; 4 Department of Medicine, Durham VA Health Care System, Durham NC 27705, USA; 5 Department of Pharmaceutical Chemistry, Faculty of Pharmacy, King Abdulaziz University, Alsulaymanyah, Jeddah 21589, Saudi Arabia; 6 Department of Pharmaceutical Chemistry, Faculty of Pharmacy, Al-Azhar University, Cairo 11884, Egypt; 7 Division of Hematology, The Children’s Hospital of Philadelphia, Philadelphia, PA 19104, USA

**Keywords:** hemoglobin, sickle cell disease, antisickling, nitric oxide, oxygen equilibrium curve, antiadhesion, aromatic aldehydes, crystal structure

## Abstract

Sickle cell disease (SCD) is caused by a single-point mutation, and the ensuing deoxygenation-induced polymerization of sickle hemoglobin (HbS), and reduction in bioavailability of vascular nitric oxide (NO), contribute to the pathogenesis of the disease. In a proof-of-concept study, we successfully incorporated nitrate ester groups onto two previously studied potent antisickling aromatic aldehydes, TD7 and VZHE039, to form TD7-NO and VZHE039-NO hybrids, respectively. These compounds are stable in buffer but demonstrated the expected release of NO in whole blood in vitro and in mice. The more promising VZHE039-NO retained the functional and antisickling activities of the parent VZHE039 molecule. Moreover, VZHE039-NO, unlike VZHE039, significantly attenuated RBC adhesion to laminin, suggesting this compound has potential in vivo RBC anti-adhesion properties relevant to vaso-occlusive events. Crystallographic studies show that, as with VZHE039, VZHE039-NO also binds to liganded Hb to make similar protein interactions. The knowledge gained during these investigations provides a unique opportunity to generate a superior candidate drug in SCD with enhanced benefits.

## 1. Introduction

Sickle cell disease (SCD) is caused by a single-point mutation in the β-chain of the oxygen transport protein hemoglobin (Hb), where βGlu6 in normal Hb (HbA) is changed to βVal6 to form sickle Hb (HbS). Under hypoxic condition and/or when HbS molecules become deoxygenated, they polymerize into long, rigid fibers, causing sickling of red blood cells (RBCs) [1,2,3,4]. The polymer or fiber is further stabilized by several secondary interactions between the HbS molecules [5,6,7,8,9,10,11,12,13]. Consistently, naturally occurring mutations that disrupt some of the secondary interactions are known to destabilize the polymer and prevent sickling [3,5,8,9,10,11,12,13]. An example is Hb Stanleyville, where in addition to the pathogenic SCD mutation (βVal6), αAsn78 on a surface-located αF-helix of Hb is mutated to Lys, resulting in decreased HbS polymerization tendency and RBC sickling, accompanied by diminished disease severity [11,12,13].

Sickled RBCs are very rigid and brittle, causing impaired blood flow and hemolysis, which leads to a cascade of downstream adverse effects, including decreased NO bioavailability, adhesion of RBCs to vascular endothelium, hemolysis, oxidative stress, inflammation. These changes may promote painful vaso-occlusive crises (VOC) and, eventually, chronic organ damage that ultimately leads to poor quality of life and premature death [14,15,16,17,18]. Currently, there are four FDA-approved treatments for SCD in the US. L-glutamine (Endari), approved in 2017, increases the amount of NADH in RBC, helping to maintain homeostasis during oxidative stress [19,20]. Endari did not get approval from the European regulatory body due to limited evidence of efficacy in Phase III trials [20]. Crizanlizumab (Adakveo), approved in 2019, is a monoclonal antibody that targets P-selectin to prevent endothelial adhesion of erythrocytes and leukocytes, and reducing the frequency of painful VOCs by 45% [21]. However, it is unclear if this level of VOC reduction (~1 per year) is sufficient to improve quality of life. In addition, monoclonal antibodies are expensive to produce and require monthly visits to an infusion clinic, which may limit uptake. The antisickling agent, voxelotor (Oxbryta, aka GBT440), the first of a new class of aromatic aldehydes that prevents deoxygenation-induced HbS polymerization, was approved in 2019 [22,23,24]. Other aromatic aldehydes, e.g., vanillin and 5-HMF, have also been studied [3,25,26,27,28]; the later underwent phaseI/II clinical studies for the treatment of SCD but terminated due in part to poor oral bioavailability [1,2,3]. The aromatic aldehydes form Schiff-base interactions with the Hb α-subunit N-terminal αVal1 amines, inducing an allosteric transformation that stabilizes the non-polymer forming R-state oxyHbS, increasing the concentration of the non-polymer forming oxyHbS [2,3,22,23,24,25,26,27,28,29,30,31,32,33]. Finally, hydroxyurea (HU), which works by inducing the expression of fetal Hb (HbF), is the most proven therapeutic approach for SCD as demonstrated by its sustained clinical use for over two decades [34]. The induction of HbF production modulates clinical severity by directly inhibiting HbS polymerization. However, a reported lack of response to HU in up to 30% of patients and poor compliance tend to limit its use [35,36].

VZHE039 is a next-generation aromatic aldehyde that, in addition to the O_2_-dependent antisickling mechanism of Voxelotor or 5-HMF, also uniquely and directly destabilizes polymer formation, preventing RBC sickling through an O_2_-independent antisickling mechanism [2,3,32]. The latter mechanism is due to VZHE039 interacting with the protein αF-helix, which has been suggested to lead to disruption of a key intermolecular polymer contact, consequently destabilizing the polymer [32]. Several analogs of VZHE039, including TD-7, have also been studied for their antisickling activities [30,32]. Even though TD-7 shows significant antisickling activity under hypoxic conditions (O_2_-dependent antisickling mechanism), it only exhibits weak antisickling effect under anoxic condition (O_2_-independent antisickling mechanism) when compared to VZHE039, due to weak interaction with the αF-helix [32].

Free NO and other organic forms of NO, e.g., nitrate, nitrite, protein-bound S-nitrosothiols (SNOs), perform an important physiological function in human health and disease [37,38]. Conversely, the free plasma Hb from hemolyzed RBC is a potent NO scavenger, leading to NO deficiency in blood plasma, a serious pathological condition in SCD that potentiates VOC, hemolysis, inflammation, pulmonary hypertension, oxidative stress, and tissue damage [39,40,41]. In early studies, inhaled NO had positive effects in SCD patients by reducing the severity and duration of HbS-induced VOCs [42,43], and NO was also reported to reduce morbidity and mortality in an SCD mouse model [44]. Despite these promising reports, however, a Phase II study of inhaled NO found no improvement in VOC events [45], likely due to the fact that inhaled NO is not a dependable method to deliver NO systemically, and little bioavailable NO reaches the vasculature in SCD. Examples of exogenous NO-donor molecules studied for their therapeutic effects in both SCD mouse models and SCD patients include HU and L-arginine [41,46,47,48,49,50,51]. The in vivo metabolism of HU to release NO may account for HU-induced vasodilation, inhibition of platelet aggregation, and decreased inflammation activities [51,52]. Other NO-releasing compounds that have been studied either in individuals with SCD or in mouse models include L-citrulline, a non-essential amino acid that is endogenously converted to L-arginine in vivo [53]. statins, specifically simvastatin and atorvastatin [54,55,56,57]. sapropterin (BH4), polynitroxy-albumin, nitroxic-albumin, S-nitrosocysteine, Deta-NONOate, thalidomides, aspirins, pyrazoles, and indoles [41,46,47,48,49,50,58,59,60,61]. 

**Figure 1:**
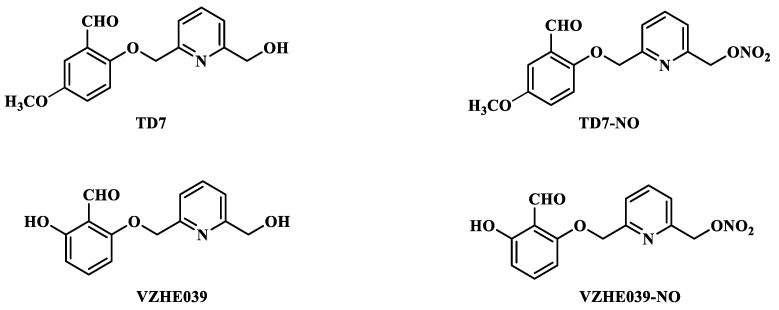
Structures of TD7, VZHE039 and their respective NO-releasing derivatives.

Despite the potential benefits of NO and/or its derivatives in SCD, several challenges have precluded their development into effective therapeutics, most prominently the capacity to deliver a therapeutic dose of bioavailable NO. In this study, we have combined into one compound an NO therapeutic modality with the unique antisickling properties of two previously studied antisickling aromatic aldehydes, TD7 and VZHE039, forming the corresponding nitrate esters TD7-NO and VZHE039-NO, respectively (Figure 1). TD7-NO and/or VZHE039-NO were first evaluated in vitro and in vivo for their ability to release NO, followed by their capability to form an adduct with Hb (Hb modification), increase Hb affinity for oxygen, and prevent deoxygenation-induced sickling *in vitro*. Further, VZHE039-NO was studied for its atomic interaction with Hb, ability to prevent SS RBCs adhesion in vitro, ability to form an adduct with Hb and to modify the protein oxygen-binding properties in vivo. 

## 2. Results

### 2.1. Chemistry

**Scheme 1:**
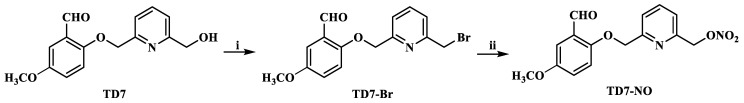
Synthesis of TD7-NO. Reagents and conditions: (i) PPh_3_, CBr_4_, anhydrous CH_2_Cl_2_, room temperature, 2 h; (ii) AgNO_3_, anhydrous CH_3_CN, 80 °C, 2 h.

**Scheme 2:**
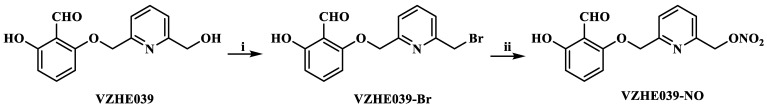
Reagents and conditions: (i) CBr_4_, PPh_3_, anhydrous DCM, r.t.; (ii) AgNO_3_, anhydrous acetonitrile, reflux at 80 °C.

In this study, we incorporated a NO-donor group onto our previously studied antisickling compounds, TD7 and VZHE039, to give the corresponding nitrate ester prodrugs TD7-NO and VZHE039-NO, following Scheme 1 and Scheme 2, respectively. To prepare TD7-NO, briefly, a solution of TD7 was reacted with triphenylphosphine and tetrabromomethane in RT to yield TD7-Br, which was converted to the nitrate ester using silver nitrate at 80 °C to give TD7-NO in 69% yield.

To prepare VZHE039-NO, briefly, a solution of VZHE039 was reacted with triphenylphosphine and carbon tetrabromide in RT to yield VZHE039-Br, which was converted to the nitrate ester using silver nitrate at 80 °C under reflux for 4 h to give VZHE039-NO in 81% yield.

### 2.2. Nitrate Ester %Derivatives of TD7 (TD7-NO) and VZHE039 (VZHE039-NO) Are Capable of Releasing NO 

**Figure 2:**
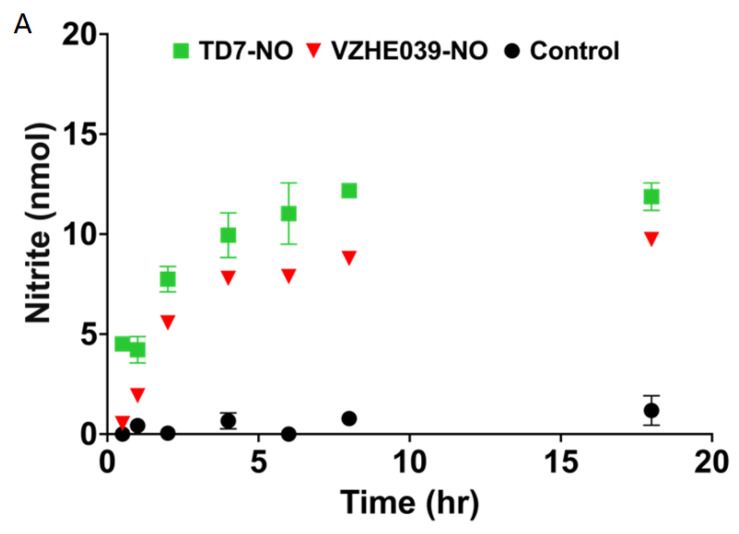

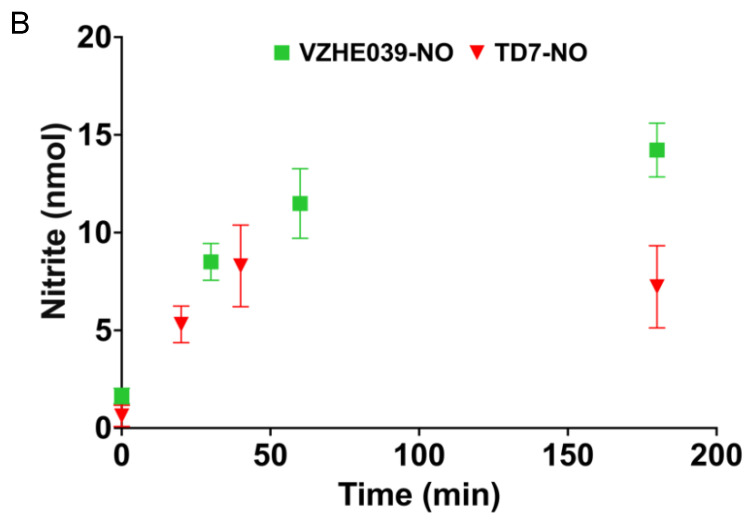
**(A**) In vitro time-dependent NO-release from VZHE039-NO and TD7-NO (1 mM). (**B**) In vivo time-dependent NO-release from VZHE039-NO and TD7-NO (IP; WT mice; 150mg/kg; n = 3).

The key to the pharmacologic effectiveness of the nitrate esters TD7-NO and VZHE039-NO would be their ability to release NO and the corresponding parent aromatic aldehydes for their respective beneficial effects. We therefore used Griess assay to measure the NO release from TD7-NO and VZHE039-NO in vitro using human whole blood and/or in vivo using wild-type mice. As expected, the in vitro study showed quicker release of the NO as compared to the in vivo study. The study also showed that both nitrate ester compounds demonstrated sustained release of NO in vitro (Figure 2A). 

Even though the in vivo experiment showed both compounds are capable of releasing NO, that of TD7 showed significant decrease after 60 min while release of NO from VZHE039 was sustained throughout the 3 h experiment (Figure 2B). This observation is presumably due to VZHE039 demonstrating favorable pharmacokinetics compared to the short-acting TD7 as a result of TD7 susceptibility to fast oxidative metabolism that transforms the active aldehyde into inactive carboxylic acids and shortens the duration of its biological activities [32]. As expected, the control compound VZHE039 exhibited no release of NO. 

### 2.3. VZHE039-NO Exhibits Favorable Pharmacologic Effect In Vitro and In Vivo

**Figure 3:**
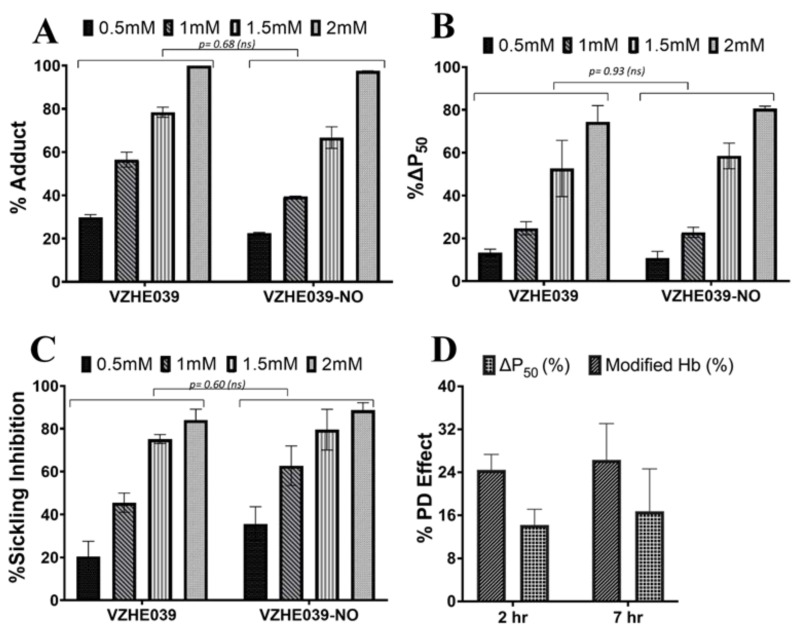
In vitro studies in SS blood showed dose-dependent effects with VZHE039 and VZHE039-NO for **(A**) Hb adducts, **(B**) P_50_ shift, and **(C**) RBC sickling inhibition. (**D**) In vivo Hb adducts and P_50_ shift of VZHE039-NO (IP; WT mice: 150 mg/kg; n = 3). The *p*-values show no statistical difference between the effects of the two molecules.

Both TD7 and VZHE039 have already been shown to be highly potent in binding to Hb and preventing deoxygenation-induced sickling [30,32]. Following the NO release studies that showed VZHE039-NO to have superior NO release compared to TD7-NO PK properties in whole blood, we further studied VZHE039-NO potential in forming adduct with Hb to increase the protein affinity for oxygen and prevent deoxygenation-induced sickling in vitro. The study conducted using SS blood showed that both VZHE039-NO and the positive control VZHE039 exhibited similar Hb modification levels, P_50_ shifts, and antisickling effects, suggesting that the NO-hybrid retains the biological properties of the parent drug (Figure 3A–C). Consistent with the in vitro results, an in vivo PD study with VZHE039-NO using wild-type mice (C57Bl/6) (IP, 150 mg/kg n = 3), showed that VZHE039-NO induced significant Hb modification and P_50_ shift of ~25% and 15%, respectively, at 2 h that was sustained through the 7 h experimental period (Figure 3D). It is clear that not only is VZHE039-NO at least as potent as VZHE039, but also shows favorable and durable pharmacologic effects.

### 2.4. VZHE039-NO Showed Antiadhesive Effect 

**Figure 4:**
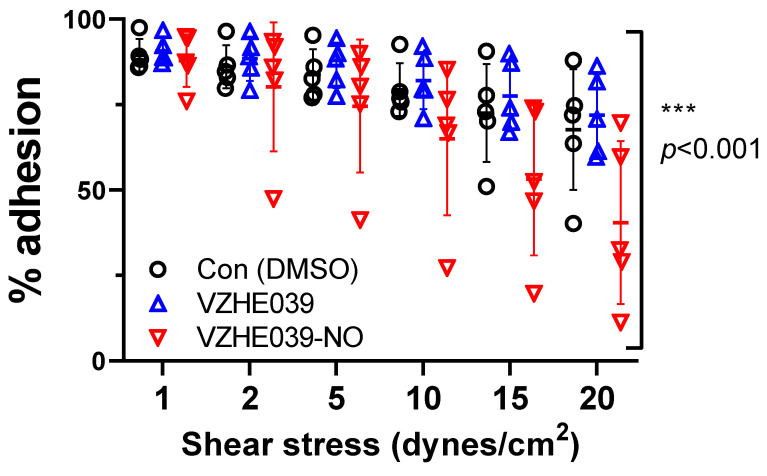
Adhesion of SS RBCs to laminin was significantly (***, *p* < 0.001 for Rx effect by mixed-effects model test) decreased by treatment with VZHE039-NO vs. either VZHE039 or vehicle alone (DMSO). Mean, SD, and individual data points are shown.

NO and its derivatives can inhibit cellular adhesion events [62,63]. We therefore performed an experiment to determine the potential of VZHE039-NO preventing adhesion of SS RBCs to immobilized laminin. Adhesion to laminin of SS RBCs treated with VZHE039 did not differ from that of vehicle-exposed SS RBCs from the same donors. By contrast, VZHE039-NO treatment significantly attenuated the adhesion of SS RBCs, as compared to either DMSO/vehicle alone or parent VZHE039 alone (*p* < 0.001 by mixed-effects model) (Figure 4). These findings are consistent with an antiadhesive effect of inclusion of the NO moiety on VZHE039. We performed a second series of experiments with additional patient samples and using another microfluidics platform (ibidi), with similar relative findings.

(1) $$\Delta \;p50\;\left(\%\right)=\frac{p50\;\mathrm{of}\;\mathrm{Control}\;\mathrm{Sample}\;–\;p50\;\mathrm{of}\;\mathrm{Test}\;\mathrm{Sample}}{p50\;\mathrm{of}\;\mathrm{Control}\;\mathrm{Sample}}\times 100$$

(2) $$\mathrm{Sickling}\;\mathrm{Inhibition}\;\left(\%\right)=\frac{\mathrm{Sickling}\;\mathrm{in}\;\mathrm{Control}\;\mathrm{Sample}\;–\;\mathrm{Sickling}\;\mathrm{in}\;\mathrm{Test}\;\mathrm{Sample}}{\mathrm{Sickling}\;\mathrm{in}\;\mathrm{Control}\;\mathrm{Sample}}\times 100$$

### 2.5. VZHE039-NO Binds Hb Similarly as VZHE039 to Stabilize Liganded Hb

As with the parent VZHE039 compound, two molecules of VZHE039-NO bind to the α-cleft of Hb, each molecule forming a Schiff base interaction between its aldehyde moiety and the N-terminal αVal1 amine in a symmetry-related fashion with the two α-subunits. The bound molecules are actually a mixture of VZHE039-NO and the hydrolyzed parent VZHE039 molecule in a ratio of 30 to 70%, both making similar interactions with the protein. The binding mode of the parent VZHE039 molecule is exactly the same as the previously published VZHE039 interactions with Hb [32], and therefore only the VZHE039-NO interactions will be discussed in detail here. The Schiff-base is strengthened by a hydrogen-bond interaction (2.5–2.6 Å) between the benzaldehyde hydroxyl and the αVal1 amine. In addition to the Schiff-base interactions, the molecules also make several intra- and inter-subunit interactions with the two α-subunits that are expected to stabilize the non-polymer-forming liganded HbS molecules and increase Hb affinity for oxygen. The hydroxyl group on the benzaldehyde ring makes inter-subunit hydrogen-bond interaction with the hydroxyl of αSer131 (3.3–3.4). The benzaldehyde ring also makes intra- and inter-hydrophobic interactions with αSer138 and αThr134, respectively. The nitro-pyridine moiety as a whole also makes intra-subunit hydrophobic interactions with both αPro77 and αMet76. It should be noted that αMet76 and αVal73 are part of the αF-helix that is known to stabilize the polymer. The interactions between VZHE039-NO and the αF-helix are expected to perturb the helix and lead to direct polymer destabilization as shown for VZHE039 [32]. Finally, there are extensive hydrophobic interactions between the two bound VZHE039-NO molecules, especially the pyridine rings that are expected to stabilize the compound’s binding to Hb. As noted above, these interactions are all conserved in VZHE039, but while VZHE039-NO uses the nitro group to interact with the αF-helix, VZHE039 uses the methylhydroxyl group. Summarily, both compounds bind to liganded Hb and make similar interactions with the protein, which should lead to similar functional/biological effect.

## 3. Discussion

Although the primary pathogenesis of SCD involves deoxygenation-induced polymerization of HbS and sickling of RBCs, the concomitant reduction in bioavailability of vascular nitric oxide (NO) due to scavenging of NO in the vasculature by free heme contributes to the pathogenesis of the disease [2,39,40]. However, while there is considerable evidence supporting the therapeutic benefits of NO, especially from NO-donor compounds for SCD, no suitable oral drug is yet available. Aromatic aldehydes have been studied extensively for their ability to prevent the primary deoxygenation-associated pathophysiology of the disease [2,3,22,23,24,25,26,27,28,29,30,31,32,33]. The HOPE clinical trial and subsequent approval of voxelotor for the treatment of SCD provided encouraging evidence that aromatic aldehydes do indeed have disease-modifying potential that not only prevents HbS polymerization but potentially can mitigate the downstream adverse disease effects of RBC sickling, such as hemolysis. While voxelotor is exclusively dependent on modulating O_2_ affinity, HbS modified by some other aromatic aldehydes, e.g., VZHE039, PP14, and to a smaller extent by TD7, resist sickling not only due to increased O_2_ affinity, but also via weakened polymer contacts that are critical to the stability of insoluble fibers (i.e., in a manner similar to HbF tetramers) [2,3,32,33]. Insight into the basis for this novel property of O_2_-independent antisickling activity comes from individuals who have a rare double mutant Hb variant, referred to as HbS Stanleyville II, possessing both the classic HbS (βGlu6→βVal6) mutation and an amino acid substitution (αAsn78→αLys78) on the surface of the Hb α-chain F-Helix [11,12,13]. Similar to individuals with hereditary persistence of HbF, inheritance of this variant results in a very benign disease [11,12,13].

In this study, we incorporated a NO-donor group onto TD7 and VZHE039, to give the corresponding nitrate ester prodrugs TD7-NO and VZHE039-NO, respectively (Figure 1). We expect that derivatives of these aromatic aldehydes that release nitric oxide on metabolism not only could restore physiologic circulating levels of NO to the vasculature but also provide the synergistic benefit of preventing HbS polymerization and/or directly destabilizing the polymer. The increased bioavailability of NO is also expected to mitigate several downstream pathophysiological events, including RBC adhesion to the vessels and the concomitant VOC. When tested in vitro (using human whole blood) and/or in vivo (using wild-type mice), the nitrate esters indeed were able to release NO (Figure 2). It is notable that these compounds are stable in DMSO at room temperature for extended periods of time, suggesting that the NO release is facilitated in the whole blood. The mechanism of biotransformation of the nitrate esters to release NO has been proposed to involve both an enzymatic action and a chemical hydrolysis [64,65,66].

Follow-up in vitro PD tests with SS blood with VZHE039-NO showed it to induce similar Hb modification levels, P_50_ shifts, and antisickling effects as the parent VZHE039, suggesting that the NO-hybrids retain the parent compound’s functional and antisickling properties (Figure 3A–C). In vivo study with WT mice (IP, 150 mg/kg n = 3) showed VZHE039-NO to induce significant Hb modification and P_50_ shift of ~25% and 15%, respectively, at 2 h that were sustained through the 7 h experimental period (Figure 3D). It is expected that, in vivo, NO is released, and that the P_50_ shift would be effected by VZHE039, consistent with the previously reported studies of RSR13-NO hybrid, an NO delivery by low-O_2_-affinity allosteric effector of Hb [64].

One of the primary focuses of this study is to determine whether aromatic aldehydes with NO releasing ability can attenuate adhesion of SS RBC in addition to its antisickling activity as described above. Indeed, in the study with SS RBC, VZHE039-NO was able to attenuate adhesion of SS RBCs, while VZHE039, as with the vehicle-exposed SS RBCs from the same donors, did not show significant attenuation of adhesion of SS RBCs (ANOVA) (Figure 4). These findings are consistent with an antiadhesive effect of inclusion of the NO moiety on VZHE039.

**Figure 5:**
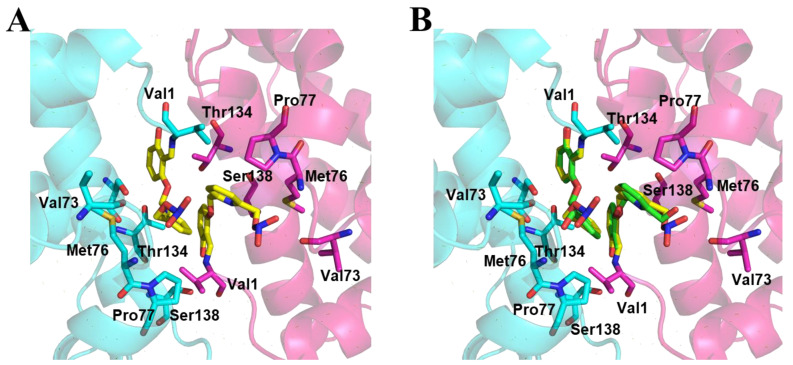
Structure of Hb in the R2 conformation in complex with two molecules of VZHE039-NO bound at the α-cleft. Note that the bound ligand is a mixture of VZHE039 and VZHE039-NO but only the bound VZHE039-NO is described. For clarity, not all binding site residues are shown. Hb subunits are shown as ribbons (α1 subunit in pink, α2 in cyan). (**A**) A pair of bound VZHE039-NO (yellow sticks) at the α-cleft of Hb showing the close interaction with the αF-helix. (**B**) Superposition of VZHE039-NO (yellow) and the previously reported VZHE-039 (green) molecules at the α-cleft of Hb.

Previous studies have elegantly elucidated how aromatic aldehydes effect their antisickling activities by binding to the relaxed state HbS, and in the presence of oxygen stabilize the R2-state Hb conformation to increase the concentration of the non-polymer-forming oxygenated HbS [2,3,22,26,27,29,30,31,32,33]. Some of these compounds, such as VZHE039 or TD7 but not Voxelotor, are also able to interact with the surface-located αF-helix to directly destabilize the polymer and prevent sickling in the absence of oxygen [3,32,33]. Although we propose that nitrate ester compounds will hydrolyze in blood to give the parent aromatic aldehydes and NO metabolites, it is quite possible that the PD effect involving Hb adduct formation, increased Hb oxygen affinity, and antisickling activity could be due to both VZHE039 and VZHE039-NO. We therefore conducted X-ray crystallographic study with liganded Hb to understand the mechanism of action of VZHE039-NO. The structure suggested a mixture of bound VZHE039-NO and VZHE039 (Figure 5), the latter presumably formed as a result of hydrolysis of VZHE039-NO by the crystallization buffer, which is known to occur during crystallization of nitrate esters [67]. The structure indicated that the intact nitrate ester compound binds similarly to VZHE039 at the α-cleft of R2-state Hb to make several interactions that stabilize the relaxed state Hb, suggesting that the intact nitrate ester molecule, as with the parent VZHE039, could prevent deoxygenation-induced sickling. Moreover, the observed close hydrogen-bond interaction between the nitrate oxygens and the αF-helix suggests that VZHE039-NO, as with the parent molecule, is also capable of directly destabilizing the polymer. Thus, it appears that VZHE039-NO, whether completely hydrolyzed to release NO and VZHE039, or partially, could still elicit favorable pharmacologic effects.

## 4. Experimental Section

### 4.1. Study Approvals

At Virginia Commonwealth University (VCU), normal whole blood (AA) was collected from healthy adult donors (>18 years) after informed consent, in accordance with regulations of the Institutional Review Board (IRB) for Protection of Human Subjects (IRB #HM1). At the Children’s Hospital of Philadelphia (CHOP), leftover blood samples from individuals with homozygous sickle cell (SS) who had not been recently transfused, were obtained and utilized based on an approved IRB protocol (IRB# 11-008151), with informed consent. At Duke University, blood samples from individuals with homozygous sickle cell (SS) who had not been recently transfused, were obtained with informed consent and utilized under an approved IRB protocol #Pro00007816. All experimental protocols and methods were performed in accordance with institutional (VCU, Duke, and CHOP) regulations. Hemoglobin for crystallographic studies was prepared from whole blood as previously published [68]. All animal experiments were performed according to the institutional ethical guidelines on animal care and approved by the Institute Animal Care and Use Committee at the Children’s Hospital of Philadelphia (approval number: IAC 19-000113). 

### 4.2. Chemistry

#### 4.2.1. Materials and General Procedures

All reagents used in the syntheses and functional assays were purchased from Sigma-Aldrich (St. Louis, MO, USA) or ThermoFisher Scientific (Waltham, MA, USA) and mostly utilized without additional purification. TD7 and VZHE039 were synthesized as previously published [30,32]. 

#### 4.2.2. Synthesis of (6-((2-Formyl-4-methoxyphenoxy)methyl)pyridin-2-yl)methyl Nitrate (TD7-NO)

Synthesis of the intermediate 2-((6-(bromomethyl)pyridin-2-yl)methoxy)-5-methoxybenzaldehyde (TD7-Br). Triphenylphosphine (140 mg, 0.6 mmol) and tetrabromomethane (180 mg, 0.6 mmol) was added to a solution of 2-((6-(hydroxymethyl)pyridin-2-yl)methoxy)-5-methoxybenzaldehyde (TD7) (100 mg, 0.4 mmol) in anhydrous CH_2_Cl_2_ (6 mL). The reaction mixture was allowed to stir for 2 h at room temperature, and then neutralized with saturated NaHCO_3_ at 0 °C (ice-bath) and extracted with EtOAc (10 mL). The organic portion was washed with brine (2 × 10 mL), dried over Na_2_SO_4_ and evaporated under reduced pressure to yield a crude product, which was purified by column chromatography (petroleum ether/EtOAc; 5:1) to yield 100 mg (74.6%) of TD7-Br as a yellow-colored solid: ^1^H-NMR (CDCl_3_): δ 3.81 (s, 3H, OCH_3_), 4.56 (s, 2H, CH_2_), 5.28 (d, 2H, CH_2_), 6.98 (d, *J* = 9.08Hz, 1H, ArH), 7.10–7.13 (dd, *J* = 9.08, 3.28 Hz, 1H, ArH), 7.33–7.37 (dd, *J* = 8.4, 3.24 Hz, 2H, ArH), 7.53 (d, *J* = 7.84 Hz, 1H, ArH), 7.80 (t, 1H, ArH), 10.58 (s, 1H, CHO). 

Synthesis of the final product (6-((2-Formyl-4-methoxyphenoxy)methyl)pyridin-2-yl)methyl nitrate (TD7-NO). Silver nitrate (76 mg, 0.45 mmol) was added to a solution of 2-((6-(bromomethyl)pyridin-2-yl)methoxy)-5-methoxybenzaldehyde (TD7-Br) (100 mg, 0.3 mmol) in anhydrous CH_3_CN (5 mL). The reaction mixture was allowed to stir at 80 °C for 2 h, filtered through celite and washed with CH_2_Cl_2_ (15 mL), and evaporated under reduced pressure to yield a crude product. The crude residue was purified by column chromatography (petroleum ether/EtOAc; 5:1) to yield 80 mg (83.78%) of TD7-NO as a white-colored solid: mp 92–93 °C; IR (KBr, cm^−1^): 1664 (C=O), 1632 (NO_2_), 1274 (NO_2_), 872 (ON); ^1^H-NMR (CDCl_3_): δ 3.81 (s, 3H, OCH_3_), 5.28 (d, 2H, CH_2_), 5.56 (s, 2H, CH_2_), 6.98 (d, *J* = 9.08Hz, 1H, ArH), 7.10–7.13 (dd, *J* = 9.08, 3.28 Hz, 1H, ArH), 7.33–7.37 (dd, *J* = 8.4, 3.24 Hz, 2H, ArH), 7.53 (d, *J* = 7.84 Hz, 1H, ArH), 7.80 (t, 1H, ArH), 10.57 (s, 1H, CHO); ^13^C-NMR (CDCl_3_): δ 189.18, 157.08, 155.20, 154.20, 152.50, 138.05, 125.52, 123.38, 121.27, 121.19, 114.85, 111.00, 74.15, 71.51, 55.85. MS (ESI) *m/z* found 319.09 (M + H)^+^, 341.07 (M + Na)^+^. The purity of the compound was checked by HPLC and was found to be 94.5% pure.

#### 4.2.3. Synthesis of (6-((2-formyl-3-hydroxyphenoxy)methyl)pyridin-2-yl)methyl Nitrate (VZHE039-NO)

Synthesis of the intermediate 2-((6-(bromomethyl)pyridin-2-yl)methoxy)-6-hydroxybenzaldehyde (VZHE039-Br). To a suspension of VZHE039 (0.1 g, 0.39 mmol) in anhydrous methylene chloride (6 mL) were added triphenylphosphine (0.253 g, 0.96 mmol) and carbon tetrabromide (0.320 g, 0.96 mmol). The resulting mixture was stirred at room temperature for 4 h. The reaction mixture was quenched by adding saturated sodium bicarbonate solution at 0 °C, followed by extraction with ethyl acetate (20 mL × 3). The combined extracts were washed with saturated brine, dried over anhydrous sodium sulfate, concentrated and further purified by column chromatography to provide VZHE039-Br as a white solid. Yield: 81%. Mp: 131.1–131.5 °C. ^1^H NMR (400 MHz, DMSO-*d*_6_) *δ*: 11.75 (s, 1H, OH), 10.42 (s, 1H, CHO), 7.89 (t, *J* = 7.72 Hz, 1H, Py-H), 7.60 (d, *J* = 7.68 Hz, 1H, Py-H), 7.55–7.51 (m, 2H, Ph-H, Py-H), 6.71 (d, *J* = 8.28 Hz, 1H, Ph-H), 6.56 (d, *J* = 8.36 Hz, 1H, Ph-H), 5.31 (s, 2H, CH_2_), 4.72 (s, 2H, CH_2_). ^13^C NMR (100 MHz, DMSO-*d*_6_) *δ*: 193.87, 162.40, 160.81, 156.27, 156.02, 138.71, 138.35, 122.94, 120.98, 110.74, 109.65, 103.44, 70.72, 34.54. IR (Diamond, cm^−1^): 3014, 2919, 1651, 1616, 1597, 1576, 1457, 1442, 1399, 1363, 1345, 1317, 1295, 1243, 1217, 1199, 1176, 1133, 1106, 1084, 993, 876, 840, 785, 742, 723, 673. ESI-MS: *m/z* 343.9905 [M + Na]^+^, C_14_H_12_BrNO_3_ (321.0001).

Synthesis of the final product (6-((2-formyl-3-hydroxyphenoxy)methyl)pyridin-2-yl)methyl nitrate *(VZHE039-NO)*. Silver nitrate (0.079 g, 0.47 mmol) was added to a solution of VZHE039-Br (0.1 g, 0.31 mmol) in anhydrous acetonitrile (4 mL). The reaction mixture was stirred under reflux (80 °C) for 4 h. The mixture was then filtered through celite and then washed with methylene chloride. The combined filtrate was concentrated under reduced pressure. The residue was purified by column chromatography to provide VZHE039-NO as a yellow solid. Yield: 81%. Mp: 58.9–59.3 °C. 1H NMR (400 MHz, DMSO-*d*_6_) *δ*: 11.75 (s, 1H, OH), 10.41 (s, 1H, CHO), 7.95 (t, *J* = 7.76 Hz, 1H, Py-H), 7.67 (d, *J* = 7.76 Hz, 1H, Py-H), 7.54–7.48 (m, 2H, Ph-H, Py-H), 6.70 (d, *J* = 8.12 Hz, 1H, Ph-H), 6.56 (d, *J* = 8.40 Hz, 1H, Ph-H), 5.70 (s, 2H, CH_2_), 5.32 (s, 2H, CH_2_). ^13^C NMR (100 MHz, DMSO-*d*_6_) *δ*: 193.85, 162.40, 160.77, 156.07, 152.34, 138.68, 138.27, 121.67, 121.50, 110.72, 109.65, 103.40, 74.24, 70.60. IR (Diamond, cm^−1^): 2913, 1635, 1597, 1448, 1399, 1359, 1342, 1317, 1280, 1241, 1221, 1199, 1166, 1106, 1094, 989, 976, 913, 850, 838, 804, 770, 749, 722, 698, 669. ESI-MS: *m/z* 327.0575 [M + Na]^+^, C_14_H_12_N_2_O_6_ (304.0695).

### 4.3. Study of NO Release from the Nitrate Esters in Whole Blood In Vitro

The ability of TD7-NO and VZHE039-NO to release the bound NO when incubated with whole blood in vitro was tested in a time-dependent manner using Griess assay as reported by us in the literature for the recently studied nitrate ester of 5HMF (5HMF-NO) [67]. Briefly, whole blood from normal healthy individuals (adjusted to hematocrit of 30%) was mixed in 96 well plate (in duplicate) with the nitrate esters (and VZHE039 as a control) to a final concentration of 1 mM and incubated in a shaker at 37 °C. Aliquots of blood were withdrawn at 0.5, 1, 2, 4, 6 and 18 h, and subsequently centrifuged (in a microcentrifuge) to separate out the plasma. To 50 µL of the separated plasma, 2.5 µL *Aspergillus* nitrate reductase (0.05U), 0.25 µL FAD (5 µM) and 0.25 µL NADPH (0.1 mM) were added to convert nitrate to nitrite. A 197 µL buffer (50 mM HEPES, PH 7.5) was added to adjust the volume to 250 µL, and the tubes incubated at room temperature for 2h. Following, 2.5 µL lactate dehydrogenase (2 mg/mL) and 25 µL Na pyruvate (100 mM stock) were added to consume leftover NADPH, and the tubes further incubated at room temperature for another 15 min. Fifty µL of freshly mixed Griess reagent (Thermo Scientific) was added to one hundred and twenty-five µL of the reaction mixture, and the reaction volume was made to 1.5 mL with distilled water, and the tubes incubated in the dark for 30 min. The absorbance of the samples was read at 548 nm. Solutions of 0–100 μM sodium nitrite were used to generate a standard curve by a nitrite absorbance versus concentration under the same experimental conditions. NO released by the compounds (quantified based on the release of nitrite ion) was calculated from the standard nitrite concentration curve.

### 4.4. Study of NO Release from the Nitrate Esters in Whole Blood from Wild-Type Mice Dosed with the Compounds

TD7-NO and VZHE039-NO were also tested for their potential to release NO in vivo. In the study, both compounds were administered to wild-type mice (150 mg/kg; n = 3; intraperitoneal [IP]), and blood was drawn at different time points of 0, 30 60 and 180 min for VZHE039-NO, and 0, 20, 40 and 60 min for TD7. The released NO was determined at each time point using the procedure described above with the Griess assay. 

### 4.5. In Vitro Hb Modification, Oxygen Equilibrium and Antisickling Studies Using Human Homozygous Sickle Cell (SS) Blood

VZHE039-NO (with VZHE039 as a positive control) was studied for its in vitro pharmacodynamic effect, including ability to form adduct and modify Hb, increase Hb affinity for oxygen, and inhibit deoxygenation-induced RBC sickling as previously reported [32]. Briefly, SS blood (hematocrit of 20%) suspensions were incubated under air in the absence or presence of 0.2, 0.5, 1.0 and 2.0 mM concentration of test compound at 37 °C for 1 h. The suspensions were then incubated under hypoxic condition (2.5% O_2_/97.5% N_2_) at 37 °C for 2 h. Aliquot samples were fixed with 2% glutaraldehyde solution without exposure to air, and then subjected to microscopic morphological analysis. For the Hb modification study, hemolysates from the above antisickling studies were subjected to HPLC (Hitachi D-7000 Series, Hitachi Instruments, Inc., San Jose, CA, USA) using a weak cation-exchange column (Poly CAT A: 35 mm × 4.6 mm, Poly LC, Inc., Columbia, MD, USA) to determine adduct formation. A commercial standard consisting of approximately equal amounts of composite HbF, HbA, HbS and HbC (Helena Laboratories, Beaumont, TX, USA), was utilized as the reference for isotypes. The areas of new peaks, representing HbS adducts, were obtained, calculated as percentage fractions of total Hb area, and reported as levels of modified Hb. 

Finally, for oxygen equilibrium studies, aliquot samples from the above clarified lysate were also subjected to hemoximetry analysis using a Hemox^TM^ Analyzer (TCS Scientific Corp. New Hope, PA. USA) to determine the P_50_ values.

### 4.6. In Vivo Pharmacologic Study of VZHE039-NO in Wild-Type Mice

VZHE039-NO was studied in vivo with C57BL/6 mice for its ability to form adduct and increase Hb affinity. All animal experiments were performed according to the institutional ethical guidelines on animal care and approved by the Institute Animal Care and Use Committee at the Children’s Hospital of Philadelphia (approval number: IAC 19-000113). The animals were treated with a single intraperitoneal (IP) (150 mg/kg) dose of the compounds. Blood samples were obtained prior to treatment (0) and 2 and 7 h post-treatment, via submandibular bleeding, and subjected to hemolysis using standard methods. Clarified lysates, free of cell debris and red blood cell ghosts, were subjected to cation-exchange HPLC to determine the drug-modified hemoglobin (adducts), as well as for oxygen equilibrium studies to determine degrees of shift in P_50_ values using methodologies described earlier for in vitro studies on human samples [32]. 

### 4.7. Study of VZHE039 and VZHE039-NO on Adhesivity of SS RBCs

We quantified the adhesion of SS RBCs to immobilized laminin in the presence and absence of VZHE039 and VZHE039-NO using a Fluxion Bioflux microfluidics system. Cells from each donor were treated with VZHE039-NO or VZHE039 (2 mM), or an equivalent concentration of DMSO (0.8%, vehicle). Control (DMSO vehicle) and treated samples from a given donor were studied on the same day. Microchannels were precoated with laminin (10 µg/mL in PBS) either for two hours at room temperature or overnight at 4 °C. Cells were suspended in PBS at 10^7^ cells/mL, perfused into the microchannel at 37 °C at a flow producing a shear stress of 0.4 dynes/cm^2^, and allowed to settle and adhere for 15 min without flow. Before exposure to flow, phase-contrast images of the microchannel surface were obtained using an inverted microscope at 10X (pre-wash; shear = 0). Then, flow was initiated, and the shear stress was incrementally set to the values shown, 1–20 dynes/cm^2^, and held constant for 5 min at each shear stress. Imaging was repeated at each shear stress. Quantification of laminin-adherent RBCs was performed manually using Adobe Photoshop 2021 within the same rectangular field of view after each shear stress increment, and the area of the rectangular fields of view was constant among samples and experiment dates.

### 4.8. Crystallographic Study of Hb in Complex with VZHE039-NO

X-ray crystallography was performed to study the atomic interactions of VZHE039-NO with Hb, following previously reported procedure for determining the structure of Hb with VZHE039 [32]. Briefly, 40 mg/mL of Hb was evacuated for ~1 h to make deoxygenated Hb, followed by saturation with carbon monoxide. The resulting CO-ligated Hb (COHb) was incubated with 20 molar excess of VZHE039-NO for about 1 h to form the COHb-VZHE039-NO complex. Sodium cyanoborohydride (NaCNBH_4_) in 10 molar excess of Hb was added to reduce the reversible Schiff base adduct to the corresponding irreversible alkylamine covalent bond. Subsequent crystallization of the COHb-compound complex using the batch method resulted in cherry-red needle crystals. X-ray diffraction data were collected at 100 K using Rigaku MicroMax™ 007HF X-ray Generator, Eiger R 4M Detector and Oxford Cobra Cryo-system (The Woodlands, TX). The crystals were first cryoprotected with 80 μL mother liquor mixed with 62 μL of 50% PEG6000. The diffraction data were processed using Rigaku d*trek software (The Woodlands, TX, USA) and the CCP4 suite of programs [69]. The crystal structure of the complex was solved by a molecular replacement method with the Phenix program [70,71]. using the native R2-state crystal structure (PDB ID 1BBB) as a search model. The structure was refined using Phenix and COOT [70,71,72]. The atomic coordinates and structure factors of the structure are deposited in the RCSB Protein Data Bank as entry 8EGI.

### 4.9. Statistical Analyses

All functional and biological assays evaluating NO release, antisickling activities, Hb modification, oxygen affinity changes and adhesion properties were conducted in 2–5 biological replicates. Results are reported as mean values with standard deviations, from the replicate analyses.

## 5. Conclusions

In a proof-of-concept study, we successfully incorporated nitrate ester groups onto our previously studied antisickling aromatic aldehyde compounds TD7 and VZHE039 to form TD7-NO and VZHE039-NO hybrids, respectively. These compounds show significant NO release and/or antisickling capabilities both in vitro and in vivo and also attenuate the adhesivity of SS RBCs, an effect that might significantly decrease the frequency or severity of vaso-occlusion, to which red cell adhesion is a major contributor. This study presents a unique opportunity to generate a superior drug candidate for SCD to ameliorate the plethora of primary and secondary pathophysiologic effects, including RBC sickling, hemolysis, inflammation, oxidative stress, painful VOCs, and chronic organ damage.

## Data Availability

The atomic coordinate and structure factor files have been submitted to the Protein Data Bank under an accession code 8EGI for the VZHE039-NO complex structure.

## Sample Availability

Samples of the compounds VZHE039, VZHE039-NO and TD7-NOare available from the authors.
